# The Euro Area Periphery Debt Conundrum

**DOI:** 10.1007/s10272-022-1080-3

**Published:** 2022-10-10

**Authors:** Robin Brooks, Jonathan Pingle

**Affiliations:** Institute of International Finance, 1333 H St NW, Suite 800E, 20005-4770 Washington, DC, USA

**Keywords:** F42, H63

## Abstract

There are many reasons to reform the Stability and Growth Pact, but that reform is no panacea. This is because the euro area periphery has increasingly entered a debt conundrum.

In the wake of the COVID-19 pandemic, a discussion has begun on reforming Europe's fiscal rules. This is an important debate, and there are many good reasons to make changes. Indeed, Brooks and Fortun (2020) laid out the problems with conventional output gap estimates, which – in our view – drastically understate slack in some periphery countries. That is a critical issue for Europe's fiscal rules because output gaps are used to cyclically adjust fiscal deficits. Gap estimates that are too small, i.e. that understate slack, may therefore promote fiscal policy that is too tight.

While this issue remains important on a conceptual level and will eventually need to be addressed, it has also been overtaken by a number of recent events. The coronavirus pandemic meant that Europe's fiscal rules were suspended to permit large deficits. Debt issuance soared – for understandable reasons – and debt levels are now much higher than just a few years ago. The most important issue, however, is financing. Large budget deficits on the euro area periphery ended up being financed almost entirely by the European Central Bank (ECB), which – via quantitative easing (QE) – bought much of the periphery debt issuance. Indeed, ever since ECB QE began in early 2015, it has been the case that the ECB constitutes the most important buyer of net new debt issuance on the periphery; the COVID-19 pandemic only extended and accentuated a trend that was already building.

ECB purchases have permitted low interest rates, which has made large debt burdens manageable. But low interest rates have an adverse side effect. They drive away private buyers, who see low yield levels as incompatible with perceived risks. Indeed, foreign buyers have been cashing out of periphery debt for the past decade. It is also possible that low interest rates discourage needed structural reforms in the euro area periphery and embolden populist politicians, who come to see de facto spread control as a safety net. This is the euro periphery debt "conundrum." On the one hand, low interest rates are needed to keep things going. On the other hand, they make it harder to ever exit an equilibrium where the ECB is increasingly on the hook for deficit financing.

The solution lies not in withdrawing ECB support. That would only restart the eurozone debt crisis that had such deleterious effects a decade ago. The solution instead is to couple ECB assistance with a renewed emphasis on hard structural reforms, which will boost medium-term growth prospects and make it easier for periphery countries to withstand the global rise in yields. That means a return to conditionality and confronting politically difficult issues like Italy's segmented labor market. In the end, there is no way to get around structural reforms. They must be part of the solution for Europe.

## How we got here

During the early period of the European debt crisis, there was a tug of war between northern Europe, which emphasised the need for "structural reforms," and southern Europe, which saw these reforms as an intrusion on its sovereignty. When the ECB took its first steps to help Italy and Spain a decade ago, it was in the context of conditionality. This is clear looking at the Trichet-Draghi letter. On August 5, 2011, ECB President Jean-Claude Trichet and his designated successor Mario Draghi sent a confidential letter to the Prime Minister of Italy. The letter called for a series of economic reforms implicitly conditioning the central bank's purchases of Italian bonds, putting an end to the "trust that the Government will take all the appropriate actions" (Trichet and Draghi, 2011). Later, when the Securities Market Program (SMP) ended and the Outright Monetary Transactions (OMT) program was announced, the ECB made OMT assistance conditional on a country's participation in a European Financial Stability Facility/European Stability Mechanism (EFSF/ESM) program, a way to link ECB help with conditionality and structural reforms (European Central Bank, 2012b). In fact, in the press conference announcing OMT, Draghi stated that "If the central bank were to intervene without any actions on the part of governments, without any conditionality, the intervention would not be effective and the Bank would lose its independence" (European Central Bank, 2012a). Overall, while the eurozone crisis saw the ECB take its first steps as a provider of assistance, there was a strong sense of conditionality to that role.

© The Author(s) 2022. Open Access: This article is distributed under the terms of the Creative Commons Attribution 4.0 International License (https://creativecommons.org/licenses/by/4.0/).

Open Access funding provided by ZBW – Leibniz Information Centre for Economics.

**Robin Brooks**, Institute of International Finance, Washington DC, USA.

**Jonathan Pingle**, Institute of International Finance, Washington DC, USA.

Figure 1
**Spread of 10-year sovereign bonds over Bund**
in basis points

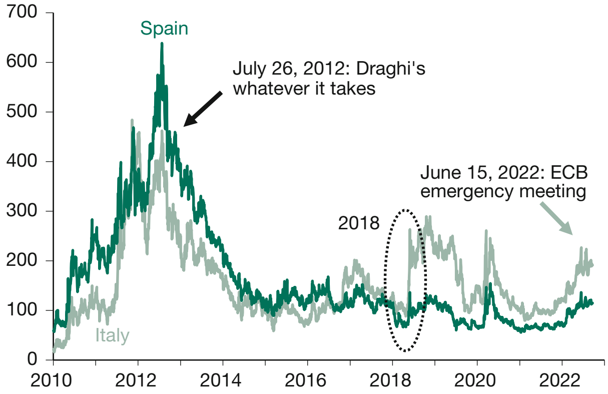

Source: Bloomberg.

Figure 2
**Standard deviation of daily changes in spread on Italian 10-year BTPs over German Bunds**
20 day rolling window

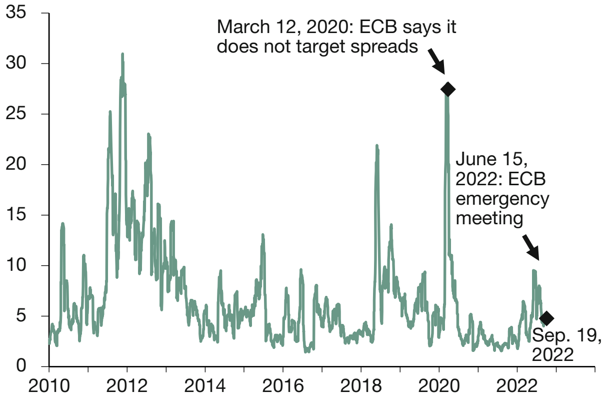

Source: Authors' calculations; Bloomberg.

The tug of war between northern and southern Europe effectively ended on July 26, 2012, when then ECB President Draghi made his now famous "whatever it takes" comment at a speech in London (European Central Bank, 2012c). That comment is certainly understandable. Market disruption in periphery bond markets was extreme at the time and people were openly speculating about a break-up of the eurozone (Figure 1). But this comment also had a material side effect. It had the unintended consequence that momentum for structural reforms – and the pressure from markets to pursue politically painful reforms – effectively ceased. What followed were years of ECB QE in the context of low inflation, which – since this was an effort to ease monetary policy on a broad basis across the eurozone – came without strings attached. While low inflation certainly justified QE, it also fed an expectation in some countries that sovereign bond purchases – without conditionality – are the norm, not the exception.

We think recent events help to put that shift into perspective. In the aftermath of the COVID-19 pandemic, global inflation has risen sharply, along with interest rates around the world. That puts pressure on highly indebted countries, given that markets tend to price higher risk premia as global rates rise. Over the course of 2022, rising risk premia widened out the spread on Italian sovereign bonds over German Bunds. That ended on June 15, 2022, with an emergency ECB meeting, which set the stage for an unveiling of the Transmission Protection Instrument (TPI) in July. While these events were unfolding, the ECB was using proceeds from maturing government bonds bought under its COVID-19 QE program to buy Italian and Spanish bonds; this was, in effect, a form of spread control. This is perhaps best visualized by looking at the volatility of Italy's spread over time. Even in the run-up to the pivotal September 2022 election, volatility is very low, an indication that some form of spread control is likely in place (Figure 2).

The pandemic has burdened the euro area periphery with higher debt levels, which – in a rising interest rate environment – carry the risk of absorbing more and more government resources. The need for the ECB to play a role is therefore hard to dispute. The issue is more about how that support is given and whether it comes with strings attached, i.e. whether it is time to re-emphasize conditionality and structural reforms.

## Deficit funding and the ECB in the aftermath of the COVID-19 pandemic

Debt levels were already diverging in the run-up to the coronavirus pandemic. Government debt in percent of GDP was on a rising trend in Italy and Spain: flat in good times and rising in bad ones. Large deficits during the pandemic exacerbated this trend, shifting debt levels materially higher. Germany's debt brake has made it an outlier. Before 2020, German debt-to-GDP levels were on a consistent downward trend (see Figure 3) and – even with large deficits during the pandemic – are no higher now than a decade ago. A similar trend can be found in the average maturity of debt. Many countries have worked to extend the average residual maturity of outstanding government debt as global interest rates have fallen. Italy is below where it was a decade ago (Figure 4), a sign that issuance gravitated to shorter maturities due to weak demand at longer maturities.

This weak demand situation is reflected in the composition of demand for net new debt issuance by governments. The bulk of funding for euro area periphery issuance in the past decade has come from the ECB. When the ECB announced

Figure 3
**General government gross debt**
in % GDP

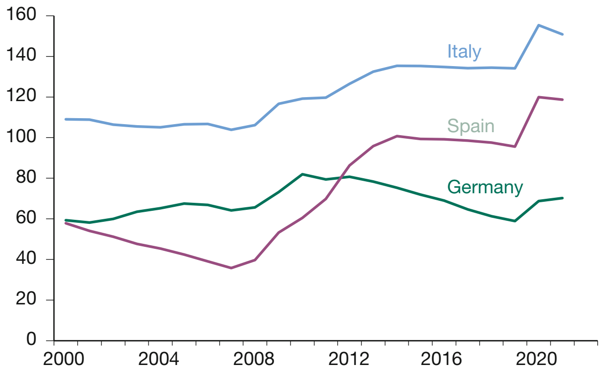

Source: IMF fiscal monitor, April 2022.

Figure 4
**Average residual maturity of government debt**
in years

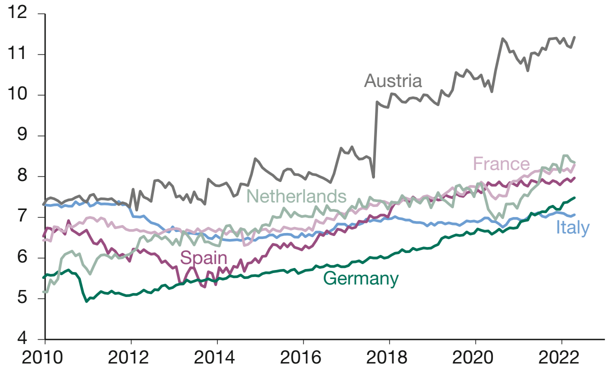

Source: European Central Bank.

the expanded asset purchase program to include sovereign bonds in early 2015, inflation was low and had remained low until recently (European Central Bank, 2015). With European inflation currently at its highest levels in history, the solidified dependence on ECB funding has become an issue.

Figures 5 and 6 highlight the issuance of government debt versus demand by sector for Italy and Spain, respectively. As shown, the public sector purchase program (PSPP) and purchases during the pandemic have accounted for most of the demand for government debt in these two countries. During these periods, there has been little demand and even outflows from foreign and domestic private investors. Before ECB QE, new issuance was demanded by a healthy mix of sectors, while at lower yields, it has become dominated by ECB demand.

Figures 7 and 8 show the same issue in debt level terms. They show that net new issuance has been absorbed entirely by the ECB over the past decade, while foreign demand and demand from domestic sources has been weak. The overall picture is therefore that low yields can be somewhat deceptive. Yields are low, but that is due almost entirely to ECB buying, not strong private sector demand. The euro area periphery debt conundrum is therefore how to bring private investors back as a

Figure 5
**Issuance of Italian government bonds vs demand by sector**
in % GDP 4-quarter moving average

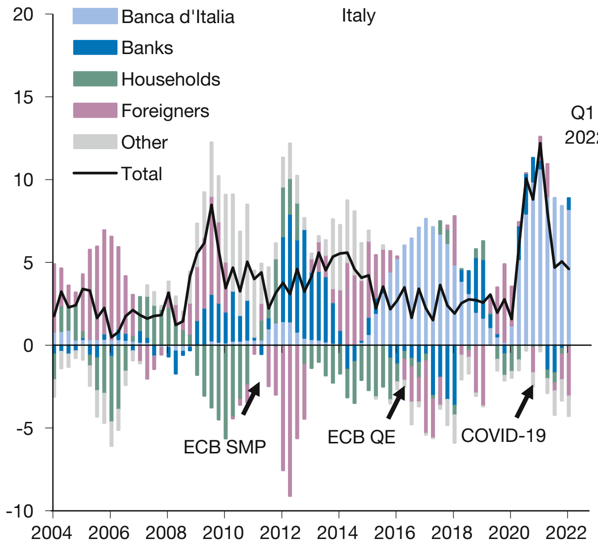

Source: Authors' calculations; Bank of Italy.

Figure 6
**Issuance of Spanish government bonds vs demand by sector**
in % GDP 4-quarter moving average

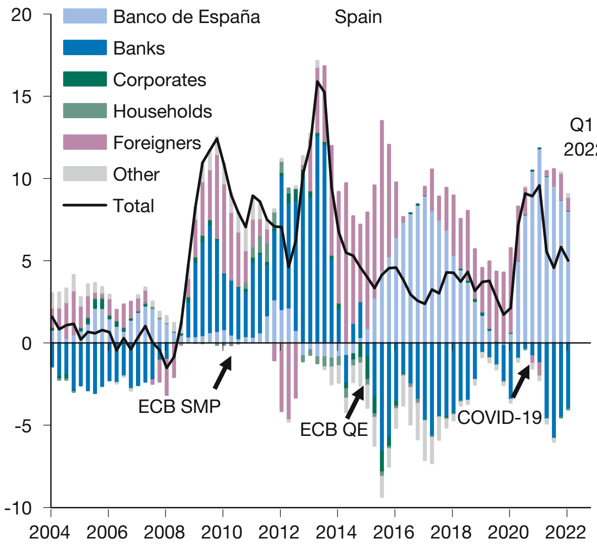

Source: Authors' calculations; Bank of Spain.

Figure 7

**Italian government debt by holder**

in % GDP

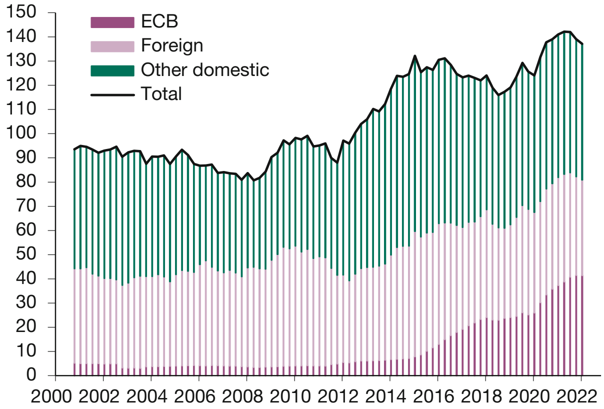

Source: Authors' calculations; Bank of Italy.

Figure 8

**Spain government debt by holder**

in % GDP

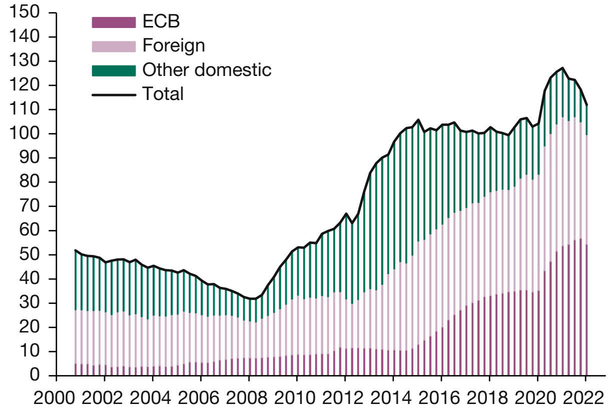

Source: Authors' calculations; Bank of Spain.

source of demand for net new issuance. That is something the ECB cannot do alone. Only a reemphasis on structural reforms can achieve that.

## Weak foreign demand as the canary in the coal mine

As the least encumbered investors, not bound by domestic regulations, foreign investors have an easier time entering and exiting. When foreign investors do not like the yield level relative to perceived risk, they leave. Jalles (2018, 397), looking at the euro area, found that "improved fiscal positions, systemic stress and financial volatility, a strong business cycle position, all increase share of public debt held by non-residents." Foreign ownership of euro area periphery debt has been falling, especially for Italy and Greece (see Figures 9 and 10). This lack of foreign demand – by investors who are less encumbered than domestic ones – is a canary in the coal mine. Low yield levels are only a temporary solution, if – one day – the goal for the ECB is to step back from sovereign bond buying.

Figure 9

**Foreign ownership of government bonds, Q3 2011 vs. Q4 2021**

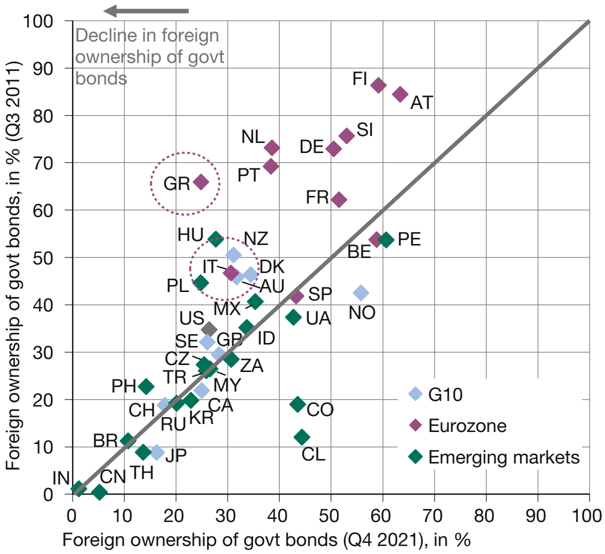

Source: IMF Sovereign Debt Investor Base.

Figure 10

**Foreign ownership of government debt across all advanced economies**

in %

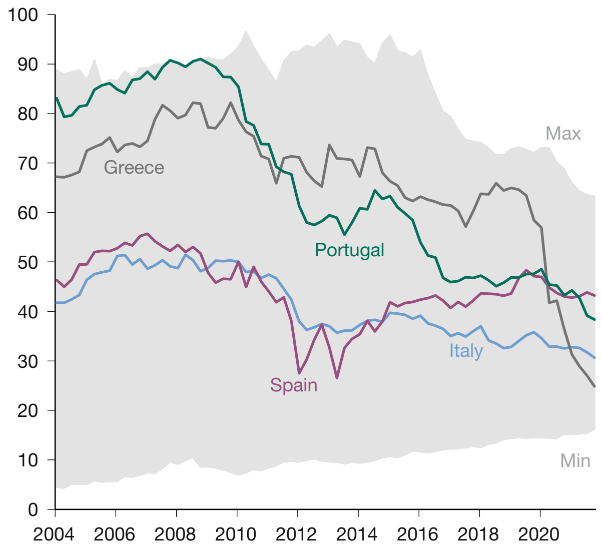

Note: Last data point: Q4 2021.

Source: IMF Sovereign Debt Investor Base

Figure 11
**Bloomberg index for liquidity in government bonds**

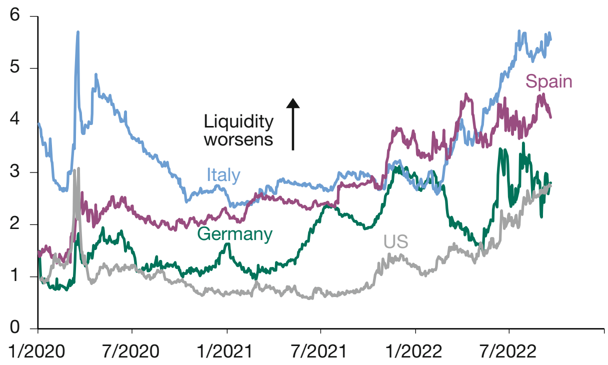

Source: Bloomberg.

Figure 12
**Monthly ECB QE purchases of sovereign bonds**

in billion euros

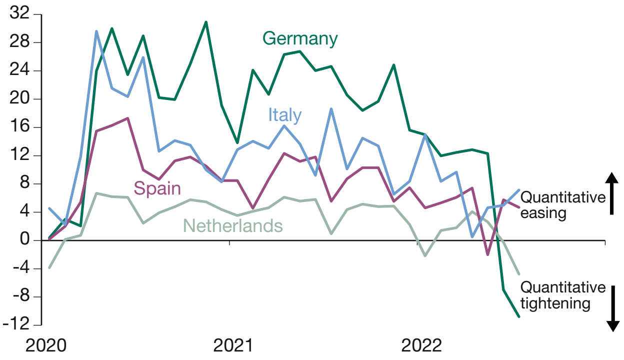

Notes: Last data point: July 2022.

Source: European Central Bank.

The rise in inflation in the aftermath of the COVID-19 pandemic is unearthing these vulnerabilities. Euro area periphery spreads have widened, especially Italian sovereign bonds over Bunds, as noted earlier (Figure 1). The deteriorating health of the bond market is especially visible in the decline in bond market liquidity. This decline in liquidity is measured as kinks in the yield curve relative to a theoretical, smooth yield curve, and, as displayed in Figure 11, is especially pronounced for Italy.

This deterioration in liquidity is happening despite continued, large ECB purchases of Italian sovereign bonds, even after QE has ended (Figure 12). This is due to reinvestments of maturing bonds bought under the pandemic emergency purchase programme (PEPP). Given the size of PEPP purchases, numerous bonds – especially for Germany – are maturing, which the ECB is using to purchase Italian debt. That is helping to keep the Italian spread well anchored, even in the run-up to the pivotal September 2022 election in Italy.

## Conclusions

There are many reasons to reform the Stability and Growth Pact, but that reform is no panacea. This is because the euro area periphery has increasingly entered a debt conundrum. It needs low interest rates for debt to be sustainable, which the ECB provides via purchases in one form or another. But low interest rates reduce the urgency for reform, with the risk that the periphery does not exit this equilibrium.

Events in mid-2022 bear out this conundrum. When Italy's spread rose modestly in the early part of the year, an emergency ECB meeting was called. Subsequent events, such as the unveiling of the TPI and large PEPP reinvestments, have kept a lid on yields, but have also – arguably – stymied any debate on what is needed to exit this state of affairs. That was especially notable in the run-up to Italy's election, where "lo spread" essentially played no role. One would expect, after so many years of ECB sovereign bond buying, an eagerness to exit this equilibrium. There is not.

We do not advocate an end to ECB sovereign bond buying. That would take the eurozone back to the dark days of the periphery debt crisis in 2011-12. We do think, however, that the eurozone needs a plan to get out of the debt conundrum. If low yields depend entirely on ECB buying, that means the ECB is permanently on the hook for sovereign bond purchases. Indeed, in the context of recent reinvestments, those purchases are only for a few countries, notably Italy. Over the medium term, the risk is that this is politically unsustainable and will breed resentment in northern Europe, where voters value a clear separation of monetary and fiscal policy. The only way to exit this conundrum is to pursue growth-enhancing reforms. Structural reform is the way to do that.
